# Supplementary material for: Influence of Stirring Parameters on Creaminess of Spring Blossom Honey Measured by Crystal Size, Whiteness Index and Mouthfeel
Source: Foods. 2022 Dec 22;12(1):48. doi: 10.3390/foods12010048 (PMC9818981; doi:10.3390/foods12010048)
Supplement: Supplementary file 1 [file foods-12-00048-s001.zip › Supplementary_information_figure_S1.pdf]

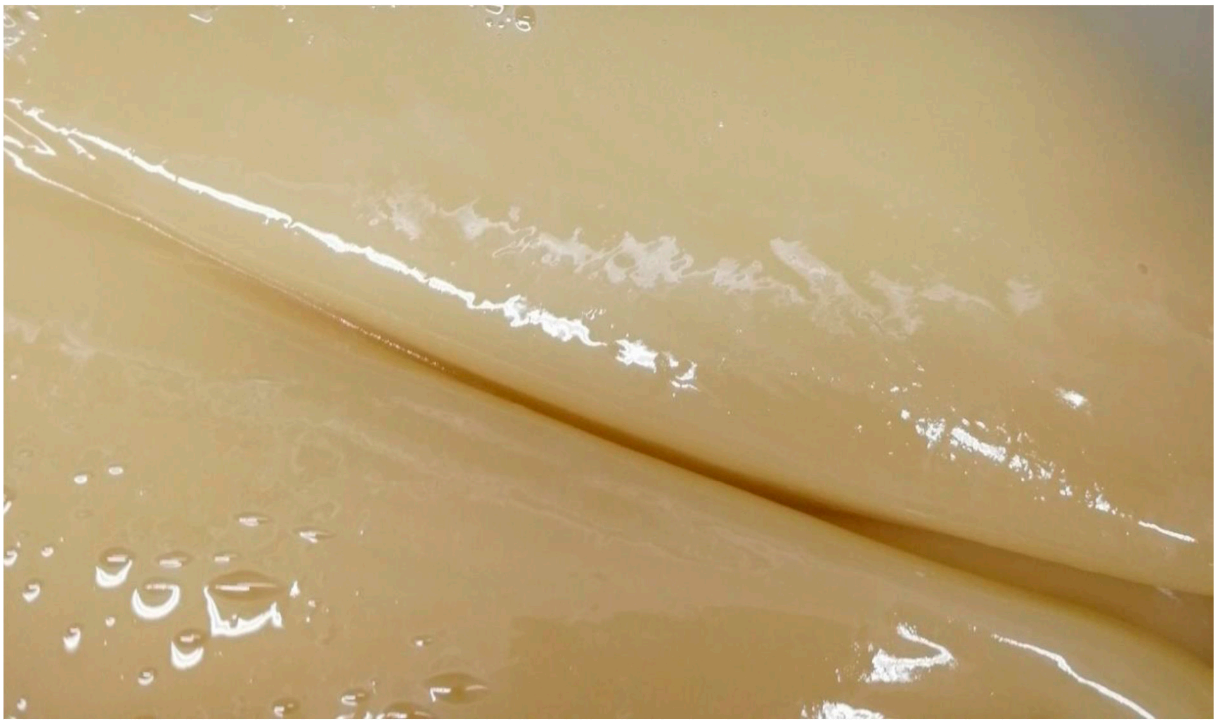

Figure S1 Modified version of the "finger test" commonly used by beekeepers. A plastic tongue depressor dipped 2 cm deep into the honey and pulled 10 cm forms two honey walls. The walls touch each other for a distance of approx. 5 cm after 5 seconds.
