# Supplementary material for: Influence of Stirring Parameters on Creaminess of Spring Blossom Honey Measured by Crystal Size, Whiteness Index and Mouthfeel
Source: Foods. 2022 Dec 22;12(1):48. doi: 10.3390/foods12010048 (PMC9818981; doi:10.3390/foods12010048)
Supplement: Supplementary file 1 [file foods-12-00048-s001.zip › Supplementary_information_figure_S3.pdf]

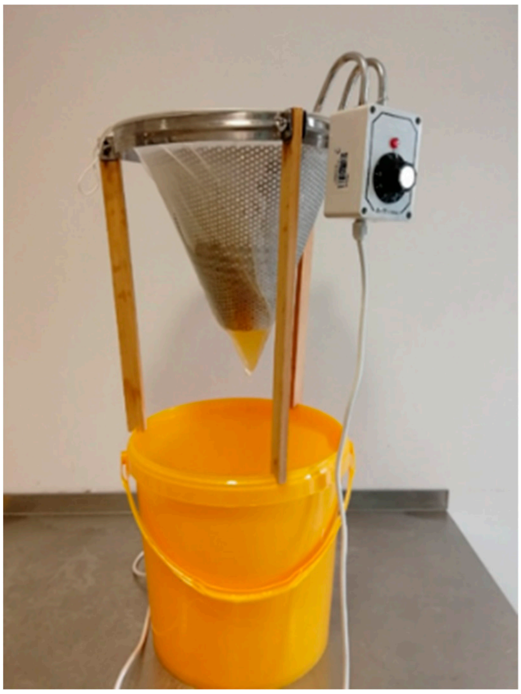

Figure S3. Prepreparation of the honey before stirring by conic pointed sieve (mesh size 200 µm) and heating coil.
