# Supplementary material for: Influence of Stirring Parameters on Creaminess of Spring Blossom Honey Measured by Crystal Size, Whiteness Index and Mouthfeel
Source: Foods. 2022 Dec 22;12(1):48. doi: 10.3390/foods12010048 (PMC9818981; doi:10.3390/foods12010048)
Supplement: Supplementary file 1 [file foods-12-00048-s001.zip › supplementary_information_table_S1_Significance_analysis_of_the_findings.pdf]

Table S1: Significance analysis of the findings (Student-Newman-Keuls procedure)

(a) Difference of the size of the 10 largest crystals in honey stirred with different stirring devices 3 days after stirring end

Stirring device	Stirring screw	Stirring impeller	Stirring spiral	Manual stirring
Not stirred	sig. ($\alpha = 0.05$)	-	-	sig. ($\alpha = 0.05$)
Stirring screw		sig. ($\alpha = 0.05$)	sig. ($\alpha = 0.05$)	-
Stirring impeller			-	sig. ($\alpha = 0.05$)
Stirring spiral				sig. ($\alpha = 0.05$)

(b) Difference of the size of the 10 largest crystals in honey stirred with different stirring devices 7 days after stirring end

Stirring device	Stirring screw	Stirring impeller	Stirring spiral	Manual stirring
Not stirred	-	-	-	-
Stirring screw		-	-	-
Stirring impeller			-	-
Stirring spiral				-

(c) Difference of the size of the 10 largest crystals in honey stirred with different stirring devices 14 days after stirring end

Stirring device	Stirring screw	Stirring impeller	Stirring spiral	Manual stirring
Not stirred	-	-	-	-
Stirring screw		-	-	-
Stirring impeller			-	-
Stirring spiral				-

(d) Difference of the size of the 10 largest crystals in honey stirred with different stirring devices 56 days after stirring end

Stirring device	Stirring screw	Stirring impeller	Stirring spiral	Manual stirring
Not stirred	-	-	-	-
Stirring screw		-	-	-
Stirring impeller			-	-
Stirring spiral				-

(e) Difference of the whiteness index of honey stirred with different stirring devices at stirring end

Stirring device	Stirring screw	Stirring impeller	Stirring spiral	Manual stirring
Not stirred	sig. (α = 0.05)	sig. (α = 0.05)	sig. (α = 0.05)	sig. (α = 0.05)
Stirring screw		-	-	-
Stirring impeller			-	-
Stirring spiral				-

(f) Difference of the whiteness index of honey stirred with different stirring devices 3 days after stirring end

Stirring device	Stirring screw	Stirring impeller	Stirring spiral	Manual stirring
Not stirred	sig. (α = 0.05)	sig. (α = 0.05)	sig. (α = 0.05)	sig. (α = 0.05)
Stirring screw		sig. (α = 0.05)	sig. (α = 0.05)	-
Stirring impeller			-	-
Stirring spiral				-

(g) Difference of the whiteness index of honey stirred with different stirring devices 7 days after stirring end

Stirring device	Stirring screw	Stirring impeller	Stirring spiral	Manual stirring
Not stirred	-	-	-	-
Stirring screw		-	-	-
Stirring impeller			-	-
Stirring spiral				-

(h) Difference of the whiteness index of honey stirred with different stirring devices 14 days after stirring end

Stirring device	Stirring screw	Stirring impeller	Stirring spiral	Manual stirring
Not stirred	-	-	-	-
Stirring screw		-	-	-
Stirring impeller			-	-
Stirring spiral				-

(i) difference of the whiteness index of honey stirred with different stirring devices 56 days after stirring end

Stirring device	Stirring screw	Stirring impeller	Stirring spiral	Manual stirring
Not stirred	sig. (α = 0.05)	sig. (α = 0.05)	-	sig. (α = 0.05)
Stirring screw		sig. (α = 0.05)	sig. (α = 0.05)	sig. (α = 0.05)
Stirring impeller			sig. (α = 0.05)	-
Stirring spiral				sig. (α = 0.05)

(j) Difference of the size of the 10 largest crystals in honey pretreated with different methods on stirring day 1

Pretreatment	Sieve, no heating coil	No sieve, no heating coil
Sieve & heating coil	sig. (α = 0.05)	sig. (α = 0.05)
Sieve, no heating coil		-

(k) Difference of the size of the 10 largest crystals in honey pretreated with different methods on stirring day 5

Pretreatment	Sieve, no heating coil	No sieve, no heating coil
Sieve & heating coil	sig. (α = 0.05)	sig. (α = 0.05)
Sieve, no heating coil		-

(l) Difference of the size of the 10 largest crystals in honey pretreated with different methods 7 days after stirring end

Pretreatment	Sieve, no heating coil	No sieve, no heating coil
Sieve & heating coil	sig. (α = 0.05)	sig. (α = 0.05)
Sieve, no heating coil		sig. (α = 0.05)

(m) Difference of the size of the 10 largest crystals in honey pretreated with different methods 14 days after stirring end

Pretreatment	Sieve, no heating coil	No sieve, no heating coil
Sieve & heating coil	sig. (α = 0.05)	sig. (α = 0.05)
Sieve, no heating coil		sig. (α = 0.05)

(n) difference of the size of the 10 largest crystals in honey pretreated with different methods 28 days after stirring end

Pretreatment	Sieve, no heating coil	No sieve, no heating coil
Sieve & heating coil	sig. (α = 0.05)	sig. (α = 0.05)
Sieve, no heating coil		-

(o) Difference of the whiteness index of honey pretreated with different methods on stirring day 1

Pretreatment	Sieve, no heating coil	No sieve, no heating coil
Sieve & heating coil	-	-
Sieve, no heating coil		-

(p) Difference of the whiteness index of honey pretreated with different methods on stirring day 5

Pretreatment	Sieve, no heating coil	No sieve, no heating coil
Sieve & heating coil	-	-
Sieve, no heating coil		-

(q) Difference of the whiteness index of honey pretreated with different methods 7 days after stirring end

Pretreatment	Sieve, no heating coil	No sieve, no heating coil
Sieve & heating coil	-	sig. ($\alpha = 0.05$)
Sieve, no heating coil		sig. ($\alpha = 0.05$)

(r) Difference of the whiteness index of honey pretreated with different methods 14 days after stirring end

Pretreatment	Sieve, no heating coil	No sieve, no heating coil
Sieve & heating coil	-	sig. ($\alpha = 0.05$)
Sieve, no heating coil		sig. ($\alpha = 0.05$)

(s) Difference of the whiteness index of honey pretreated with different methods 28 days after stirring end

Pretreatment	Sieve, no heating coil	No sieve, no heating coil
Sieve & heating coil	-	-
Sieve, no heating coil		-

(t) Difference of the size of the 10 largest crystals in honey stirred at different temperatures and pre-treated with different seeding at stirring end

Stirring temp. and seeding	14 °C with seed honey	18 °C no seed honey	18 °C with seed honey	Room temp. no seed honey
14 °C no seed honey	-	sig. (α = 0.05)	-	sig. (α = 0.05)
14 °C with seed honey		sig. (α = 0.05)	-	sig. (α = 0.05)
18 °C no seed honey			sig. (α = 0.05)	sig. (α = 0.05)
18 °C with seed honey				sig. (α = 0.05)

(u) Difference of the size of the 10 largest crystals in honey stirred at different temperatures and pre-treated with different seeding 14 days after stirring end

Stirring temp. and seeding	14 °C with seed honey	18 °C no seed honey	18 °C with seed honey	Room temp. no seed honey
14 °C no seed honey	sig. (α = 0.05)	sig. (α = 0.05)	sig. (α = 0.05)	sig. (α = 0.05)
14 °C with seed honey		sig. (α = 0.05)	sig. (α = 0.05)	sig. (α = 0.05)
18 °C no seed honey			sig. (α = 0.05)	sig. (α = 0.05)
18 °C with seed honey				sig. (α = 0.05)

(v) Difference of the size of the 10 largest crystals in honey stirred at different temperatures and pre-treated with different seeding 28 days after stirring end

Stirring temp. and seeding	14 °C with seed honey	18 °C no seed honey	18 °C with seed honey	Room temp. no seed honey
14 °C no seed honey	-	-	sig. (α = 0.05)	sig. (α = 0.05)
14 °C with seed honey		-	sig. (α = 0.05)	sig. (α = 0.05)
18 °C no seed honey			sig. (α = 0.05)	sig. (α = 0.05)
18 °C with seed honey				sig. (α = 0.05)

(w) Difference of the whiteness index of honey stirred at different temperatures and pre-treated with different seeding at the end of the stirring process

Stirring temp. and seeding	14 °C with seed honey	18 °C no seed honey	18 °C with seed honey	Room temp. no seed honey
14 °C no seed honey	sig. (α = 0.05)	sig. (α = 0.05)	sig. (α = 0.05)	sig. (α = 0.05)
14 °C with seed honey		sig. (α = 0.05)	-	sig. (α = 0.05)
18 °C no seed honey			sig. (α = 0.05)	sig. (α = 0.05)
18 °C with seed honey				sig. (α = 0.05)

(x) Difference of the whiteness index of honey stirred at different temperatures and pre-treated with different seeding 14 days after stirring end

Stirring temp. and seeding	14 °C with seed honey	18 °C no seed honey	18 °C with seed honey	Room temp. no seed honey
14 °C no seed honey	-	sig. (α = 0.05)	sig. (α = 0.05)	sig. (α = 0.05)
14 °C with seed honey		sig. (α = 0.05)	sig. (α = 0.05)	sig. (α = 0.05)
18 °C no seed honey			-	sig. (α = 0.05)
18 °C with seed honey				sig. (α = 0.05)

(y) Difference of the whiteness index of honey stirred at different temperatures and pre-treated with different seeding 28 days after stirring end

Stirring temp. and seeding	14 °C with seed honey	18 °C no seed honey	18 °C with seed honey	Room temp. no seed honey
14 °C no seed honey	sig. (α = 0.05)	sig. (α = 0.05)	sig. (α = 0.05)	sig. (α = 0.05)
14 °C with seed honey		sig. (α = 0.05)	sig. (α = 0.05)	sig. (α = 0.05)
18 °C no seed honey			sig. (α = 0.05)	sig. (α = 0.05)
18 °C with seed honey				sig. (α = 0.05)

(z) Difference of the size of the 10 largest crystals in honey stirred in different intervals at stirring end

Stirring interval	Daily twice 37,5min	Daily 4 times 18,3 min	Daily 6 times 12,5 min	Daily 24 times 3,1 min
Daily once 75 min	-	-	-	-
Daily twice 37,5min		-	-	-
Daily 4 times 18,3 min			-	-
Daily 6 times 12,5 min				-

(aa) difference of the whiteness index of honey stirred in different intervals at stirring end

Stirring interval	Daily twice 37,5min	Daily 4 times 18,3 min	Daily 6 times 12,5 min	Daily 24 times 3,1 min
Daily once 75 min	-	-	sig. ($\alpha = 0.05$)	sig. ($\alpha = 0.05$)
Daily twice 37,5min		-	sig. ($\alpha = 0.05$)	sig. ($\alpha = 0.05$)
Daily 4 times 18,3 min			-	sig. ($\alpha = 0.05$)
Daily 6 times 12,5 min				sig. ($\alpha = 0.05$)

(ab) difference of the whiteness index of honey stirred in different intervals 23 days after stirring end

Stirring interval	Daily twice 37,5min	Daily 4 times 18,3 min	Daily 6 times 12,5 min	Daily 24 times 3,1 min
Daily once 75 min	-	-	-	-
Daily twice 37,5min		-	-	-
Daily 4 times 18,3 min			-	-
Daily 6 times 12,5 min				-

(ac) Difference of the size of the 10 largest crystals in honey stirred in different durations at the end of stirring

Stirring time	Daily once 15 min	Daily twice 1 min	Daily twice 5 min	Daily twice 15 min
Daily once 1 min	-	sig. (α = 0.05)	-	sig. (α = 0.05)
Daily once 15 min		sig. (α = 0.05)	-	sig. (α = 0.05)
Daily twice 1 min			sig. (α = 0.05)	sig. (α = 0.05)
Daily twice 5 min				sig. (α = 0.05)

(ad) Difference of the size of the 10 largest crystals in honey stirred in different durations 30 days after stirring end

Stirring time	Daily once 15 min	Daily twice 1 min	Daily twice 5 min	Daily twice 15 min
Daily once 1 min	-	-	-	sig. (α = 0.05)
Daily once 15 min		-	-	sig. (α = 0.05)
Daily twice 1 min			-	-
Daily twice 5 min				sig. (α = 0.05)

(ae) Difference of the whiteness index of honey stirred in different durations at the end of stirring

Stirring time	Daily once 15 min	Daily twice 1 min	Daily twice 5 min	Daily twice 15 min
Daily once 1 min	sig. (α = 0.05)	-	-	sig. (α = 0.05)
Daily once 15 min		sig. (α = 0.05)	sig. (α = 0.05)	sig. (α = 0.05)
Daily twice 1 min			-	sig. (α = 0.05)
Daily twice 5 min				sig. (α = 0.05)

(af) Difference of the whiteness index of honey stirred in different durations 8 days after stirring

Stirring time	Daily once 15 min	Daily twice 1 min	Daily twice 5 min	Daily twice 15 min
Daily once 1 min	-	-	-	-
Daily once 15 min		-	-	sig. (α = 0.05)
Daily twice 1 min			-	-
Daily twice 5 min				-
